# Primary hypothyroidism with growth failure and pituitary pseudotumor in a 13-year-old female: a case report

**DOI:** 10.1186/1752-1947-7-149

**Published:** 2013-05-31

**Authors:** Noelle S Larson, Jordan E Pinsker

**Affiliations:** 1 Department of Pediatrics, Division of Pediatric Endocrinology, Tripler Army Medical Center, 1 Jarrett White Road, Honolulu, HI 96859, USA

**Keywords:** Children, Hypothyroidism, Growth, Pituitary

## Abstract

**Introduction:**

Primary hypothyroidism is a well-known cause of poor linear growth in children. A rare finding with profound or long-standing disease is anterior pituitary enlargement (pituitary pseudotumor). This case highlights this uncommon finding, discusses clinical situations in which gradual dose escalation of levothyroxine may be advisable and reviews adjuvant therapies that have been previously attempted to improve final height in the setting of profound hypothyroidism.

**Case presentation:**

We report the case of a 13-year-old Hispanic girl initially evaluated for poor linear growth and delayed puberty, and found to have pituitary enlargement secondary to profound primary hypothyroidism. Treatment with progressive doses of levothyroxine normalized her symptoms and led to complete resolution of her pituitary findings, but she then rapidly progressed through puberty, achieving an adult height of only 142cm, significantly below her calculated mid-parental height.

**Conclusions:**

In cases of severe primary hypothyroidism with prolonged elevation of thyroid-stimulating hormone and pituitary pseudotumor, gradual replacement of thyroid hormone with slowly escalating doses of levothyroxine may be beneficial to prevent complications of therapy. Early recognition and treatment of hypothyroidism during childhood is essential for normal growth, as final height is invariably compromised in children with prolonged disease. Additional study is needed to determine the potential beneficial effects of gonadotropin-releasing hormone agonist and recombinant human growth hormone treatment in this setting.

## Introduction

Hypothyroidism is a well-known cause of poor linear growth in children [1]. When hypothyroidism is prolonged or severe, pituitary enlargement (pituitary pseudotumor) can also be present [2]. This case highlights this uncommon finding, discusses clinical situations in which gradual dose escalation of levothyroxine may be advisable and reviews adjuvant therapies that have been previously attempted to improve final height in the setting of profound hypothyroidism.

## Case presentation

A 13-year-old Hispanic girl presented to her primary care physician for concerns of poor linear growth and delayed puberty. She had only limited access to medical care and had not been seen for a well-child visit for many years. Upon initial evaluation, she was noted to have had almost no linear growth since six years of age. She complained of cold intolerance, dry skin, and was easily fatigued with physical activity. On physical examination, her height and weight were at the 50th percentile for a six-year-old (a height standard deviation score (SDS) of −5). She had no breast development. She did not have a goiter. Her skin was very dry and galactorrhea was not present.

**Figure 1:**
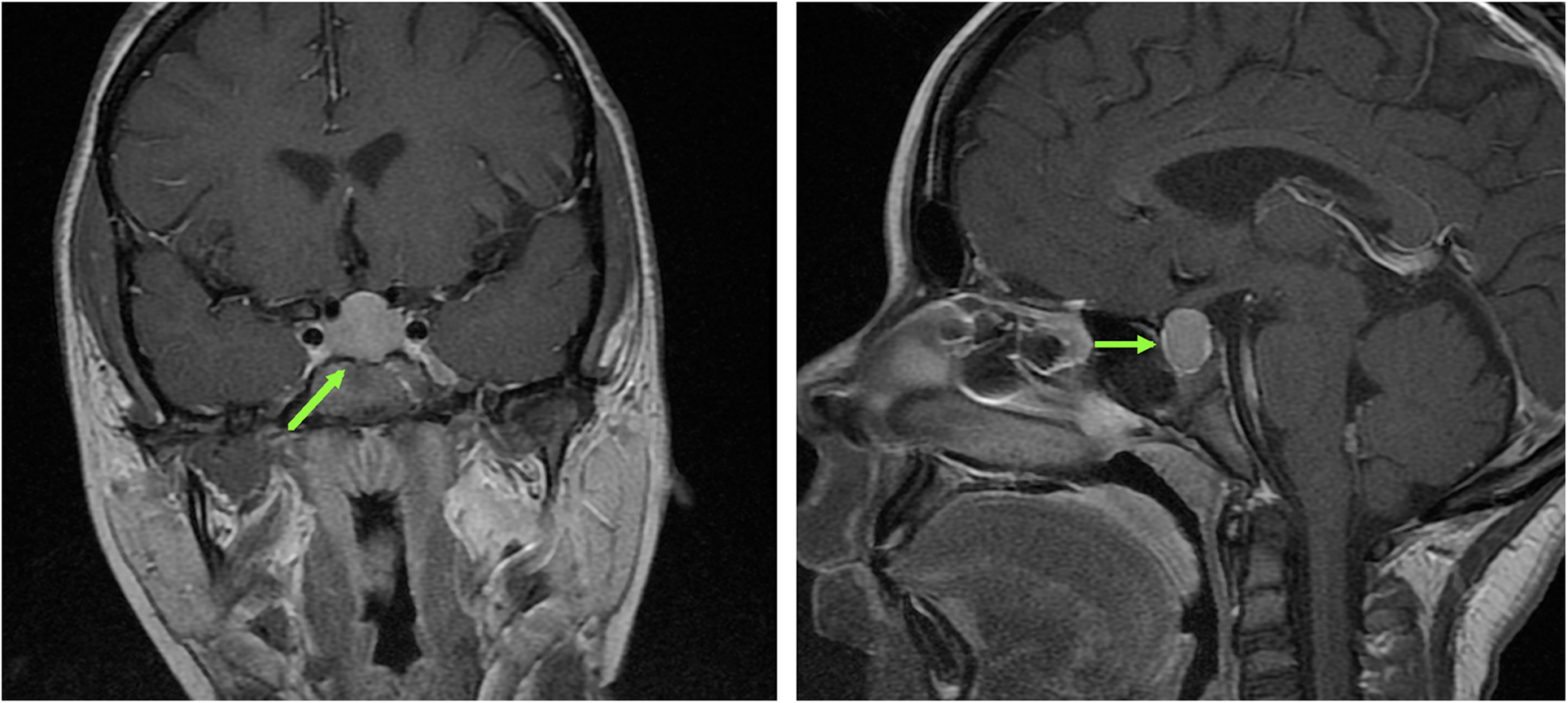
**Enlarged pituitary gland at presentation.** The enlarged pituitary gland measured 1.4cm in size and extended into the suprasellar cistern (green arrows, coronal and sagittal views on T1-weighted post-gadolinium images).

Extensive evaluation by a geneticist was undertaken due to concern for a possible syndromic cause of her poor growth. Her karyotype was normal. A brain magnetic resonance imaging (MRI) scan was also performed, which showed an enlarged pituitary gland, raising concern for a possible pituitary macroadenoma (Figure 1). The patient was then referred to the endocrinology department, where additional testing showed thyroid-stimulating hormone (TSH) > 1000mU/L with an undetectable free T4 level, suggesting the MRI finding was secondary to primary hypothyroidism. Insulin-like growth factor 1 (IGF-1) was low for her age, consistent with the well-established phenomenon of decreased growth hormone secretion in severe hypothyroidism. Her bone age was five years by the standards of Greulich and Pyle at a chronological age of 13 years. Multiple biochemical abnormalities secondary to hypothyroidism were also present (Table 1). Her serum prolactin was not measured.

**Table 1:** Laboratory values at initial presentation

TSH	> 1000mU/L (normal 0.27-4.2)
FT4	< 0.5pmol/L (normal 13-23)
Karyotype	46, XX
IGF-1	11.2nmol/L (normal 25-84)
Cholesterol	11.9mmol/L (normal 1.3-5.2)
AST	78U/L (normal 14-50)
ALT	51U/L (normal 9-52)
Creatinine	65.4μmol/L (normal 53-88)
Hemoglobin	5.0mmol/L (normal 7.4-9.9)
Creatine kinase	1178U/L (normal 30-135)
Cortisol	333.8nmol/L (normal 166-386)

TSH, thyroid-stimulating hormone; FT4, free T4; IGF-1, insulin-like growth factor 1; AST, aspartate aminotransferase; ALT, alanine aminotransferase.

**Figure 2:**
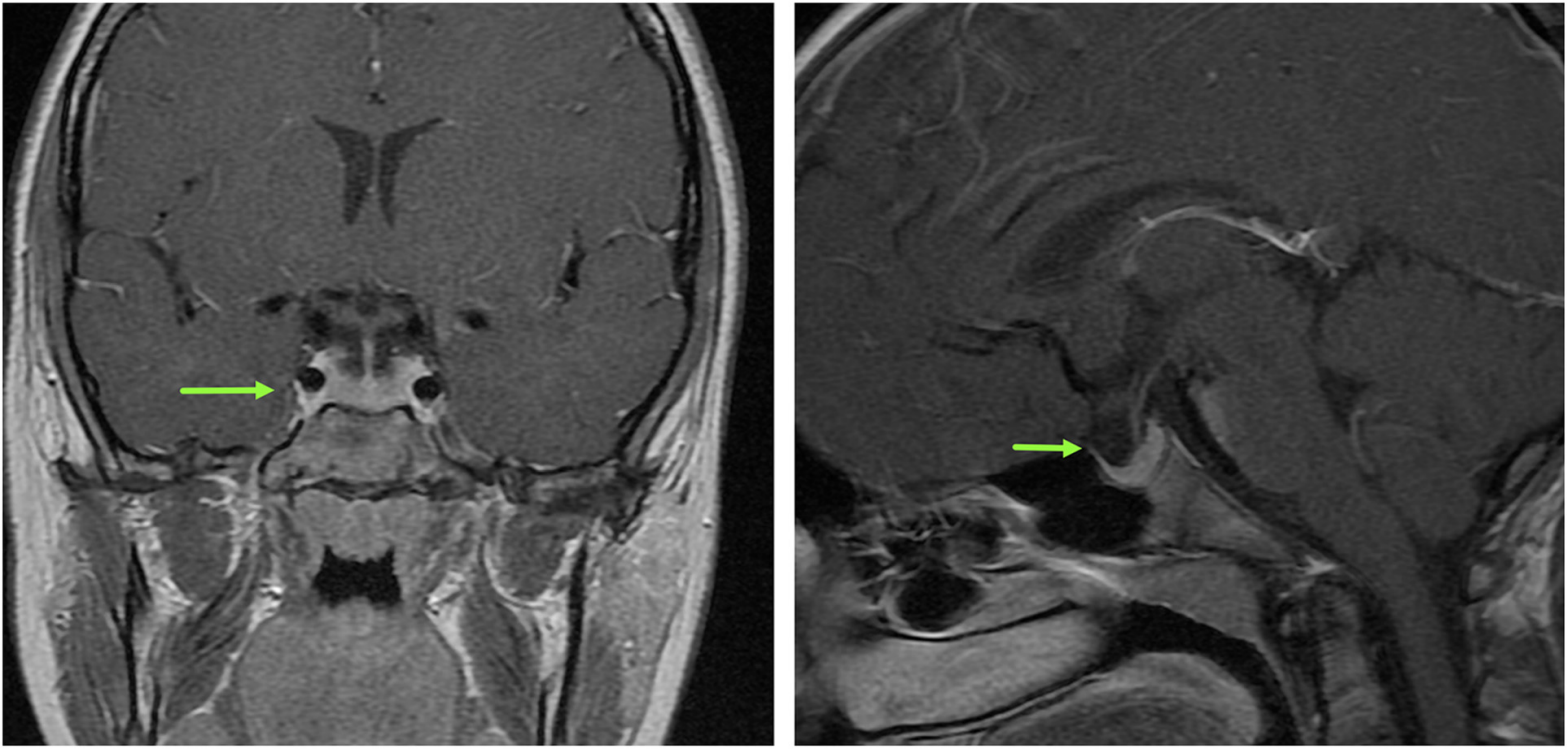
**Normal pituitary on follow-up magnetic resonance imaging scan.** Eighteen months after treatment with levothyroxine was initiated there was persistence of the enlarged sella (green arrows, coronal and sagittal views) with a normal positioned and sized pituitary gland including normal stalk and posterior bright spot (T1-weighted post-gadolinium images).

Treatment with levothyroxine was initiated at a relatively low dose of 25μg/day (1.1μg/kg/day). Over time, her dose of levothyroxine was gradually increased to 100μg/day to achieve and maintain a normal TSH level. Her linear growth velocity immediately improved to 12.9cm/year, and she rapidly progressed through puberty, achieving menarche 18 months after starting treatment. At 14 years and 10 months of age, her bone age was 12 years, indicating it had rapidly advanced. She achieved a final adult height of only 142cm (−3 SDS), significantly below her mid-parental height of 161.3cm (−0.4 SDS). A follow-up pituitary MRI scan that was done 18 months after treatment with levothyroxine was initiated showed persistence of the enlarged sella with a normal positioned and sized pituitary gland including normal stalk and posterior bright spot (Figure 2), suggesting that the enlarged pituitary seen at presentation was primarily caused by thyrotroph hyperplasia that subsequently resolved.

## Discussion

The presentation of primary hypothyroidism in children is most often marked by poor linear growth [1], although symptoms can be quite varied. In some cases, the extremely elevated thyrotropin-releasing hormone levels seen in prolonged primary hypothyroidism can lead to the rare finding of anterior pituitary enlargement (pseudotumor of the pituitary gland) [2]. Sometimes there is hyperplasia of not just thyrotrophs, but lactotrophs as well, causing hyperprolactinemia [3]. A prolactin level was not measured in our patient but hyperprolactinemia could explain in part her delayed puberty. In other cases, pituitary enlargement thought to be related to precocious puberty was actually thyrotroph hyperplasia [4,5]. In these patients, it appears that either the weak intrinsic follicle-stimulating hormone (FSH) activity of TSH can lead to cross-stimulation of the FSH receptor [6], or increased thyrotropin-releasing hormone causes an increased FSH response [7]. The true isosexual precocious puberty that results from prolonged hypothyroidism (the van Wyk-Grumbach syndrome) is unique among the causes of precocious puberty in that bone age is often delayed at presentation.

Similar to our patient, some children with extremely elevated TSH levels have been reported to present with pituitary enlargement and growth failure without goiter [8]. Goiter is a well-established feature of chronic lymphocytic thyroiditis and is often the first presenting sign of disease in children. With prolonged lymphocytic infiltration, the thyroid parenchyma may undergo fibrosis and goiter may be absent [9]. Although we did not measure thyroid peroxidase antibodies, we presume this patient had long-standing autoimmune thyroiditis.

The importance of this case is highlighted in the current controversy on treatment recommendations. Treatment with levothyroxine results in normalization of the size of the pituitary gland on subsequent imaging studies. However, in patients with severe clinical signs of hypothyroidism it may be advisable to gradually increase the dose of levothyroxine over time, rather than starting at the full anticipated replacement dose. There are a number of reasons for this. First, patients with myxedema and pericardial effusion secondary to hypothyroidism may not tolerate becoming euthyroid rapidly, potentially worsening symptoms of heart failure. This is primarily an issue for adults or children with other comorbidities, as recent reports suggest that otherwise healthy children generally tolerate more rapid replacement of thyroid hormone [10]. Second, in severe, long-standing primary hypothyroidism, it has been reported that pituitary and adrenal function may be secondarily decreased, and adrenal insufficiency may be precipitated by rapid replacement with thyroid hormone [11]. In our patient this was not an issue, as her cortisol level was normal prior to starting levothyroxine therapy. Third, there have been case reports of adults with thyrotroph hyperplasia due to primary hypothyroidism who developed empty sella following replacement therapy with levothyroxine, potentially indicating that rapid shrinkage of the enlarged anterior pituitary may have resulted in pituitary apoplexy [12]. Finally, pseudotumor cerebri has been reported as a possible rare side effect of treatment with levothyroxine.

As shown in this case, treatment with levothyroxine, even with gradually escalated doses, often rapidly advances osseous maturation, leading to premature physeal closure [7]. When combined with precocious puberty or with rapid osseous maturation following delayed puberty, there can be a significant compromise of adult height. Adjunctive therapy with gonadotropin-releasing hormone (GnRH) agonists and recombinant human growth hormone (rhGH) has been attempted to improve adult height in patients with severe, primary hypothyroidism. Although small studies and case reports have shown beneficial results of combining GnRH agonist and rhGH treatment with levothyroxine replacement [13,14], a recent retrospective review suggested neither time to euthyroidism nor use of either GnRH agonist or rhGH significantly affected height potential [15]. This suggests that despite these treatments, skeletal maturation rapidly advances. Additional study of these growth-promoting therapies is needed in the setting of severe primary hypothyroidism.

## Conclusions

Pituitary enlargement (pseudotumor of the pituitary gland) is a rare finding seen in prolonged and severe primary hypothyroidism. Early diagnosis and treatment is essential to preserve final height in children with severe primary hypothyroidism, as late diagnosis almost invariably results in decreased adult stature. Gradually increasing the dose of levothyroxine over time has been tried, and may be useful in children found to have associated pituitary hyperplasia, cardiac pathology, or adrenal insufficiency, but appears to be of limited utility with regard to height outcomes. All families should be counseled on the likelihood of attenuated final height. If severe growth retardation is present, there may be a role for GnRH agonist and/or rhGH treatment, but current efficacy data are insufficient to routinely recommend the use of these adjuvant treatments.

## Consent

Written informed consent was obtained from the patient’s legal guardian for publication of this case report and any accompanying images. A copy of the written consent is available for review by the Editor-in-Chief of this journal.

## Abbreviations

FSH: Follicle-stimulating hormone; GnRH: Gonadotropin-releasing hormone; IGF-1: Insulin-like growth factor 1; MRI: Magnetic resonance imaging; rhGH: Recombinant human growth hormone; SDS: Standard deviation score; TSH: Thyroid-stimulating hormone.

## Competing interests

The authors declare that they have no competing interests.

## Authors’ contributions

NSL provided direct patient care for this patient, and was a major contributor in writing the manuscript. JEP was a major contributor in writing the manuscript. Both authors read and approved the final manuscript.
